# Curcumin has immunomodulatory effects on RANKL‐stimulated osteoclastogenesis in vitro and titanium nanoparticle‐induced bone loss in vivo

**DOI:** 10.1111/jcmm.14842

**Published:** 2019-12-17

**Authors:** Chao Yang, Kechao Zhu, Xiangwei Yuan, Xianlong Zhang, Yebin Qian, Tao Cheng

**Affiliations:** ^1^ Department of Orthopedics Shanghai Jiao Tong University Affiliated Sixth People’s Hospital Shanghai China

**Keywords:** Akt pathway, curcumin, immunomodulatory, NF‐κB pathway, osteoclastogenesis, osteolysis

## Abstract

Wear particle‐stimulated inflammatory bone destruction and the consequent aseptic loosening remain the primary causes of artificial prosthesis failure and revision. Previous studies have demonstrated that curcumin has a protective effect on bone disorders and inflammatory diseases and can ameliorate polymethylmethacrylate‐induced osteolysis in vivo. However, the effect on immunomodulation and the definitive mechanism by which curcumin reduces the receptor activators of nuclear factor‐kappa B ligand (RANKL)‐stimulated osteoclast formation and prevents the activation of osteoclastic signalling pathways are unclear. In this work, the immunomodulation effect and anti‐osteoclastogenesis capacities exerted by curcumin on titanium nanoparticle‐stimulated macrophage polarization and on RANKL‐mediated osteoclast activation and differentiation in osteoclastic precursor cells in vitro were investigated. As expected, curcumin inhibited RANKL‐stimulated osteoclast maturation and formation and had an immunomodulatory effect on macrophage polarization in vitro. Furthermore, studies aimed to identify the potential molecular and cellular mechanisms revealed that this protective effect of curcumin on osteoclastogenesis occurred through the amelioration of the activation of Akt/NF‐κB/NFATc1 pathways. Additionally, an in vivo mouse calvarial bone destruction model further confirmed that curcumin ameliorated the severity of titanium nanoparticle‐stimulated bone loss and destruction. Our results conclusively indicated that curcumin, a major biologic component of *Curcuma longa* with anti‐inflammatory and immunomodulatory properties, may serve as a potential therapeutic agent for osteoclastic diseases.

## 1 INTRODUCTION

Total joint replacement (TJR) has achieved great success in the field of orthopedic surgery in the last few decades. TJR can reduce the pain and restore the joint function of patients with severe joint diseases.^1^, ^2^ However, wear debris‐mediated bone loss and the consequent implant loosening remain major obstacles for TJR.^3^ Studies have demonstrated that wear debris from the implant materials, including titanium particles (TiPs), ultra‐high molecular weight polyethylene particles and CoCrMo particles, enhance the production of proinflammatory cytokines and the activation of osteoclastic signalling pathways.^4^, ^5^ Finally, the balance between osteoblasts and osteoclasts becomes disrupted, resulting in pathological osteolytic diseases.

As mentioned, wear debris‐stimulated inflammatory reactions and a proinflammatory microenvironment are critical for the activation of osteoclast precursor cells and the subsequent osteoclast formation.^6^, ^7^ Therefore, inhibiting the inflammatory response and reducing the release of proinflammatory cytokines are considered an effective strategy for preventing and treating wear debris‐mediated periprosthetic osteolysis. In recent years, the immunoregulation of macrophage polarization was identified as a critical mechanism in the process of implant loosening.^8^ Wear debris‐stimulated M0 macrophages turn into proinflammatory macrophages (M1 phenotype) and subsequently produce of proinflammatory chemokines. This proinflammatory microenvironment triggers the activation of osteoclastic signalling pathways and induces the differentiation of the hematopoietic monocyte/macrophage linage. However, M2 phenotype macrophages secrete anti‐inflammatory cytokines and create an anti‐inflammatory microenvironment, which subsequently inhibit the differentiation and formation of osteoclasts.^9^, ^10^, ^11^ It has been reported that the regulation of macrophage polarization can mitigate wear debris‐stimulated osteolysis.^8^ Similar to this finding, in our previous study, we reported that curcumin ameliorated inflammatory reactions by inhibiting M1 polarization in a mouse air‐pouch model.^12^ Therefore, we have been suggested that the modulation of M1/M2 macrophages represents a potential therapeutic strategy to alleviate wear debris‐mediated osteolysis.

Curcumin, a polyphenol and a major biologic component in the extract of the root of *Curcuma longa*, exhibits biologic properties because of its anti‐inflammatory, antioxidant, anti‐tumour and antimicrobial activities.^13^, ^14^, ^15^ We have previously reported that curcumin attenuated polymethylmethacrylate‐stimulated bone destruction in mice and alleviated Ti particle (TiP)‐stimulated inflammation by modulating macrophage polarization.^12^, ^16^ In addition, previous studies have suggested that curcumin exerted a protective effect on bone disorders and inflammatory diseases, such as osteolysis, rheumatoid arthritis and osteoporosis.^17^, ^18^ Moreover, curcumin alleviates the up‐regulation of nuclear factor‐kappa B (NF‐κB) phosphorylation in bone loss, which is associated with the functional state of osteoclasts.^15^ Previous studies have shown that several osteoclastic signalling pathways, including the mitogen‐activated protein kinase (MAPK), nuclear factor‐kappa B (NF‐κB) and phosphatidylinositol 3‐kinase/AKT (PI3k/Akt) pathways, are regulated by the activation of receptor activators of nuclear factor‐kappa B ligand (RANKL) and RANK and that cross‐talk occurs in these pathways. However, the definitive mechanism by which curcumin ameliorates RANKL‐stimulated osteoclastogenesis and up‐regulates osteoclastic signalling pathways is unclear. Based on previous findings, we have been suggested that curcumin prevents the formation of an inflammatory microenvironment by regulating the ratio of M1/M2 macrophages and then further attenuates RANKL‐mediated osteoclast maturation and formation via suppressing the related osteoclastic signalling pathway.

The purpose of this study was to evaluate the immunomodulatory effects of curcumin on wear debris‐stimulated inflammatory responses and identify the definitive mechanism by which it affects osteoclastogenesis, and classical mouse and cell models were used to provide a reliable basis for clinical applications in the future. Therefore, the direct anti‐osteoclastogenesis and immunomodulatory effects of curcumin on RANKL‐stimulated osteoclast differentiation and TiP‐mediated changes in macrophage polarization in vitro were investigated. Furthermore, the responsible mechanisms at the cellular level were also explored by Western blotting. Then, micro‐computed tomography (micro‐CT) and histological staining were used to investigate the therapeutic effectiveness of curcumin in vivo in a mouse calvarial model, and immunofluorescence staining was used to evaluate macrophage polarization in vivo.

## 2 MATERIALS AND METHODS

### 2.1 Preparation of TiPs and reagents

TiPs were obtained from Johnson Matthey Chemical and autoclaved at 121°C for 15 minutes. A Limulus amebocyte lysate assay (Biowhittaker) was applied to determine the endotoxin level of TiPs, as previously described.^19^ Curcumin (purity ≥ 98.0%, MW: 368.37) was obtained from Solarbio and dissolved in dimethyl sulfoxide (2 mg/mL). α‐Minimum essential medium (α‐MEM; HyClone) and Dulbecco's modified Eagle's medium (DMEM, HyClone) containing 10% foetal bovine serum (FBS; Gibco) and 1% penicillin/streptomycin (HyClone) were used to culture the cells.

### 2.2 Osteoclast precursor cell isolation and culture

Bone marrow‐derived macrophages (BMMs) and RAW264.7 macrophages were used for in vitro experiments. BMMs were obtained from the bone marrow of 4‐week‐old male C57BL/6J mice and cultured in complete α‐MEM containing 10 ng/mL M‐CSF for 1 day. Then, the suspension cells were resuspended and incubated with 30 ng/mL M‐CSF for 3 days. The BMMs were used for further experiments at approximately 80% confluence. RAW cells were cultured in complete DMEM.

### 2.3 Cell viability assay

The BMMs (1 × 10^4^) were seeded on a 96‐well plate and cultured in complete α‐MEM containing 30 ng/mL M‐CSF for 1 day. The medium was replaced with fresh complete medium containing various concentrations of curcumin (0, 0.5, 1.25, 5, 10, 20, 30, 40, 50 or 100 μmol/L) in the next day. After culturing for 3 days, the medium was replaced with fresh complete α‐MEM containing 10% CCK‐8 solution and the cells were cultured for an additional 3 hours. A microplate reader was used to evaluate cell viability at a wavelength of 450 nm.

### 2.4 Curcumin attenuated RANKL‐mediated osteoclast maturation

Bone marrow‐derived macrophages were used to investigate the direct anti‐osteoclastogenic effect of curcumin on osteoclast formation. The cells were induced in complete medium supplemented with 30 ng/mL M‐CSF, 100 ng/mL RANKL and 0, 1.25, 5 or 20 μmol/L curcumin. In addition, BMMs were plated and induced in osteoclastic induction medium and 20 μmol/L curcumin was added at day 0, 2, or 4, respectively. After 6 days, BMMs were rinsed three times and fixed for 15 minutes. A tartrate‐resistant acid phosphatase (TRAP; Sigma) staining kit was applied to stain osteoclasts.

### 2.5 F‐actin ring formation and bone resorption area assays

To measure the functional state of osteoclasts, F‐actin ring formation and osteoclastic resorption were assessed to evaluate the inhibitory effect of curcumin. BMMs were seeded and cultured as described above. After 6 days, the cells were fixed and permeabilized. Then, the cells were stained with phalloidin and DAPI for 15 minutes to visualize the cytoskeleton and nucleus, respectively. Fluorescence microscopy (Leica) was used to observe F‐actin ring formation. In addition, BMMs were plated on an Osteo Assay Plate (OAP; Corning) and induced as described above. When mature osteoclasts were observed on day 4, the osteoclastic induction medium and 0, 1.25, 5 or 20 μmol/L curcumin were replaced and cultured for an additional 2 days. At day 6, osteoclasts were removed via sonication, and the resorption pits were observed with a light microscope (Leica). The percentage of the bone resorption areas was measured using Image‐Pro Plus software.

### 2.6 Osteoclastic‐related gene expression

Bone marrow‐derived macrophages were seeded and incubated in osteoclast induction medium containing 0, 1.25, 5 or 20 μmol/L curcumin for 5 days. Furthermore, the expression of osteoclastic‐related genes with or without curcumin pre‐treatment at different stages was also investigated. Briefly, cells (1 × 10^5^) were plated on a 6‐well plate and cultured in osteoclast induction medium with or without curcumin (20 μmol/L) for 1, 3 and 5 days. TRIzol reagent (Invitrogen) was used to extract total RNA. Then, 1μg of total RNA was used to synthesize complementary DNA using M‐MLV reverse transcriptase (Takara). SYBR Premix Ex Taq (Takara) was applied for quantitative gene analysis. Gene primers are shown in Table 1 with GAPDH as a housekeeping gene.

**Table 1:** Primers sequences used for RT‐PCR in this study

Gene	Primer sequences (F: forward; R: reverse; 5′−3′)
c‐fos	F: CCAGTCAAGAGCATCAGCAA
	R: AAGTAGTGCAGCCCGGAGTA
NFATc1	F: CCGTTGCTTCCAGAAAATAACA
	R: TGTGGGATGTGAACTCGGAA
Oscar	F: CTGCTGGTAACGGATCAGCTCCCCAGA
	R: CCAAGGAGCCAGAACCTTCGAAACT
Sema‐4A	F: TAAAGTGAATGAAACCATTTGT
	R: GTCTGTGAAATGTTTTACAGTGT
GAPDH	F: ACCCAGAAGACTGTGGATGG
	R: CACATTGGGGGTAGGAACAC

### 2.7 Immunofluorescence staining of p65 in RAW264.7 cells

The cells were added to a 24‐well plate and pretreated with or without 20 μmol/L curcumin for 4 hours and then induced with RANKL for 30 minutes. The cells were fixed, permeabilized and blocked in sequence. RAW264.7 cells were incubated with primary antibody against p65 (1:200, Cell Signaling Technologies) overnight at 4℃ and with the secondary antibody donkey anti‐rabbit Alexa Fluor 488 (1:200, Abcam) for 1 hour. DAPI was applied for 15 minutes to stain the cell nucleus.

### 2.8 Analysis of macrophage polarization in vitro by flow cytometry

To investigate the indirect anti‐osteoclastogenic effects of curcumin on osteoclast differentiation and maturation, we first evaluated the immunoregulatory activity of curcumin in RAW264.7 cells. Flow cytometry, immunofluorescence staining and ELISA were performed to confirm the regulation of macrophage polarization as previously described. RAW264.7 cells cultured without TiPs treatment were used as a negative control group, whereas cells incubated with 0.1 mg/mL TiPs alone were used as a positive control group. In addition, cells treated with TiPs and 20 μmol/L curcumin were regarded as the experimental group. Briefly, RAW264.7 (5 × 10^4^) cells were plated and incubated for 3 days. Then, the cells were collected and resuspended. Allophycocyanin (APC)‐conjugated CCR7 (eBioscience) and phycoerythrin (PE)‐conjugated CD206 (eBioscience) antibodies were used to stain the cells for 1 hour. A Guava flow cytometer was used to analyse the polarization phenotype of macrophages.

### 2.9 Analysis of macrophage polarization by ELISA and immunofluorescence staining

RAW264.7 cells were seeded and cultured as described above. The supernatant was collected and centrifuged. The tumour necrosis factor alpha (TNF‐α), interleukin (IL)‐4, IL‐6 and IL‐10 levels were examined using ELISA kits (Anogen). In addition, RAW264.7 cells were fixed and blocked as described above and then incubated with antibodies against CCR7 (M1 marker, 1:100, Abcam) and Arg‐1 (M2 marker, 1:100, Abcam) overnight at 4°C. Secondary antibodies donkey anti‐rabbit Alexa Fluor 488 (1:200, Abcam) and donkey antimouse Alexa Fluor 594 (1:200, Abcam) were applied to combine with the primary antibodies for 1 hour. Finally, DAPI was applied for 15 minutes to stain the cell nucleus.

### 2.10 Western blotting

After incubation for 2 days, RAW264.7 cells were pretreated with 20 μmol/L curcumin for 6 hours and subsequently stimulated with RANKL for 5, 15 and 30 minutes. The cells were harvested and lysed for 30 minutes on ice. Total proteins were separated and transferred to polyvinylidene fluoride membranes. The membranes were cut into protein bands and blocked for 1 hour. Primary antibodies against extracellular signal‐regulated kinase (ERK), p‐ERK, p38 mitogen‐activated protein kinase (p38), p‐P38, c‐jun N‐terminal kinase (JNK), p‐JNK, phosphatidylinositol 3‐kinase/AKT (Akt), p‐Akt, NF‐κB, p‐NF‐κB, inhibitor‐κB (IκBα), p‐IκBα, c‐fos, NFATc1 and β‐actin (Cell Signaling Technologies, USA) were added followed by incubation overnight at 4°C. The next day, the protein bands were incubated with the secondary antibodies for 1 hour and visualized using an enhanced chemiluminescence reagent (Millipore).

### 2.11 In vivo mouse calvarial osteolysis model

The animal experiment was approved by the Animal Care and Experiment Committee of Sixth People's Hospital affiliated with Shanghai Jiao Tong University. Animal care and use were conducted according to the policies of the Institutional Animal Care and Use Committee of Shanghai Jiao Tong University, the regulations for the Administration of Affairs Concerning Experimental Animals (China, 2014) and the National Institutes of Health Guide for the Care and Use of Laboratory Animals (GB14925‐2010). A total of 36 healthy C57BL/6J mice were randomly divided into three groups: (a) the control group, in which the mice were subjected to a sham operation and injected daily with phosphate‐buffered saline (PBS) intraperitoneally 2 weeks; (b) the TiPs group, in which 20 mg TiPs was placed on the calvarial surface, and the mice received daily intraperitoneal injections of PBS for 2 weeks; and (c) the TiPs + Cur group, in which the mice were subjected to the operation and 20 mg TiPs and then received intraperitoneal injections of 25 mg/kg/d curcumin for 2 weeks. A calvarial resorption model was established to assess the therapeutic effectiveness of curcumin for treating TiP‐stimulated bone destruction.^20^ Briefly, the mice were anaesthetized with 4% chloral hydrate. The head of each mouse was shaved and sterilized. Then, a 1‐cm incision was cut in the skin and cranial periosteum of the head with a scalpel. Finally, 20 mg TiPs was placed on the calvarial surface of the mice in the TiPs and TiPs + Cur groups, and the wound was closed carefully. After 2 weeks, the mice were killed, and the skulls were dissected and collected for micro‐CT and histological analyses, and the skin of head was also harvested for immunofluorescence staining to evaluate macrophage polarization in vivo. In addition, the morphology of the TiPs was observed by transmission electron microscope (TEM).

To evaluate the morphology and bone destruction of the mouse calvaria, the samples were fixed in formalin for 3 days, decalcified for 21 days and then wrapped in paraffin wax. A microtome was used to cut the samples into 5‐μm sections. The sections were deparaffinized, rehydrated and subjected to haematoxylin and eosin (H&E) and TRAP staining. The area of erosion (mm^2^), number of osteoclasts and percentage of osteoclasts per bone surface (OCs/BS, %) of the samples were evaluated using Image‐Pro Plus software. In addition, immunofluorescence staining (CCR7 and Arg‐1, Abcam) was used to assess the change in macrophage polarization in vivo.

### 2.12 Statistical analysis

All the data were analysed using SPSS 17.0 software and expressed as the mean ± standard deviation (SD). One‐way analysis of variance (ANOVA) and Student's t tests were used to evaluate the significance of differences. *P* < .05 or *P* < .01 was considered significantly different.

## 3 RESULTS

### 3.1 Curcumin attenuates the differentiation and formation of osteoclastic precursor cells without cytotoxicity

**Figure 1:**
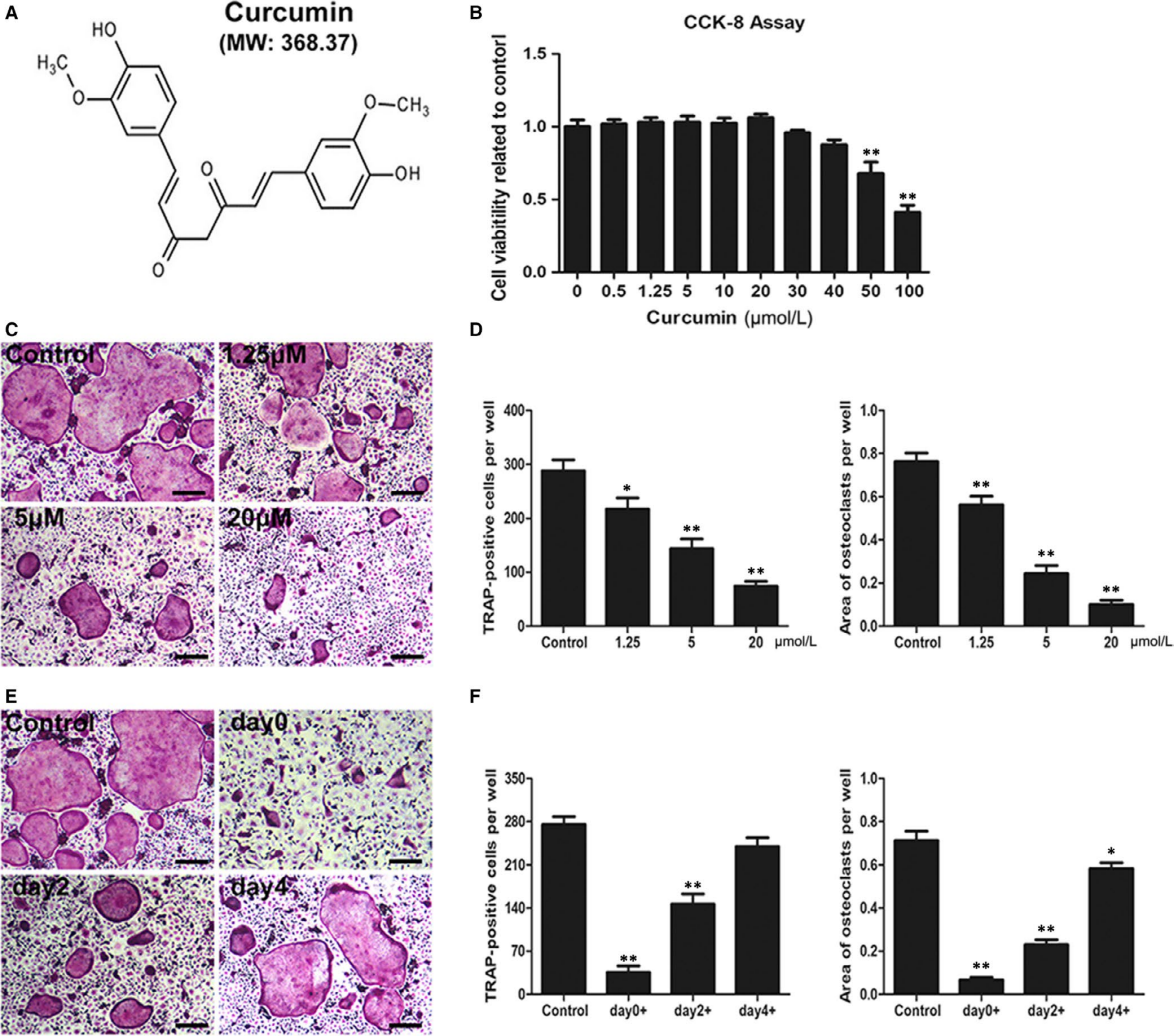
Curcumin ameliorated RANKL‐induced osteoclast differentiation and formation without cytotoxicity. A, Chemical structure of curcumin (MW = 368.37). B, Cell viability was assessed by CCK‐8 assay. C, BMMs were incubated and induced in osteoclast induction medium with various curcumin concentrations (0, 1.25, 5 and 20 μmol/L) and then subjected to TRAP staining at day 6. D, The number and area of TRAP‐positive cells under each treatment were quantified. E, BMMs were seeded and cultured in osteoclastic induction medium, and 20 μmol/L curcumin was added at day 0, 2 or 4, respectively. TRAP staining was performed at day 6. F, The number and area of TRAP‐positive cells at each time‐point were quantified. Data are presented as mean ± SD; *P < .05 and **P < .01 compared with the control group. Scale bar = 100 μm

To assess the cell toxicity of curcumin, we performed CCK‐8 assays to measure the viability of BMMs. After culturing with various curcumin concentrations for 3 days, no cytotoxicity was observed in osteoclastic precursor cells at doses below 40 μmol/L (Figure 1A,B), indicating that the protective effect of curcumin on RANKL‐mediated osteoclastogenesis was not due to cytotoxicity caused by a high dose of curcumin. We further evaluated the inhibitory effect of various curcumin concentrations (0, 1.25, 5 and 20 μmol/L) on RANKL‐mediated osteoclast formation by TRAP staining on day 6. The results of TRAP staining demonstrated that typical osteoclasts formed without curcumin intervention (0 μmol/L), whereas the number and size of osteoclasts were markedly decreased as the curcumin concentration increased (Figure 1C,D). In addition, we further performed osteoclast induction assays to identify which stages of osteoclast differentiation were affected by curcumin. Osteoclasts were significantly reduced at the early stage (days 0‐2) by treatment with 20 μmol/L curcumin; however, this early inhibitory effect was not observed at later differentiation stages (Figure 1E,F). Therefore, the osteoclast differentiation results suggested that curcumin had an inhibitory effect on RANKL‐mediated osteoclast formation in dose‐ and time‐dependent manners and this effect was not due to cytotoxicity caused by a high dose.

### 3.2 Curcumin interfered with the function of osteoclasts

**Figure 2:**
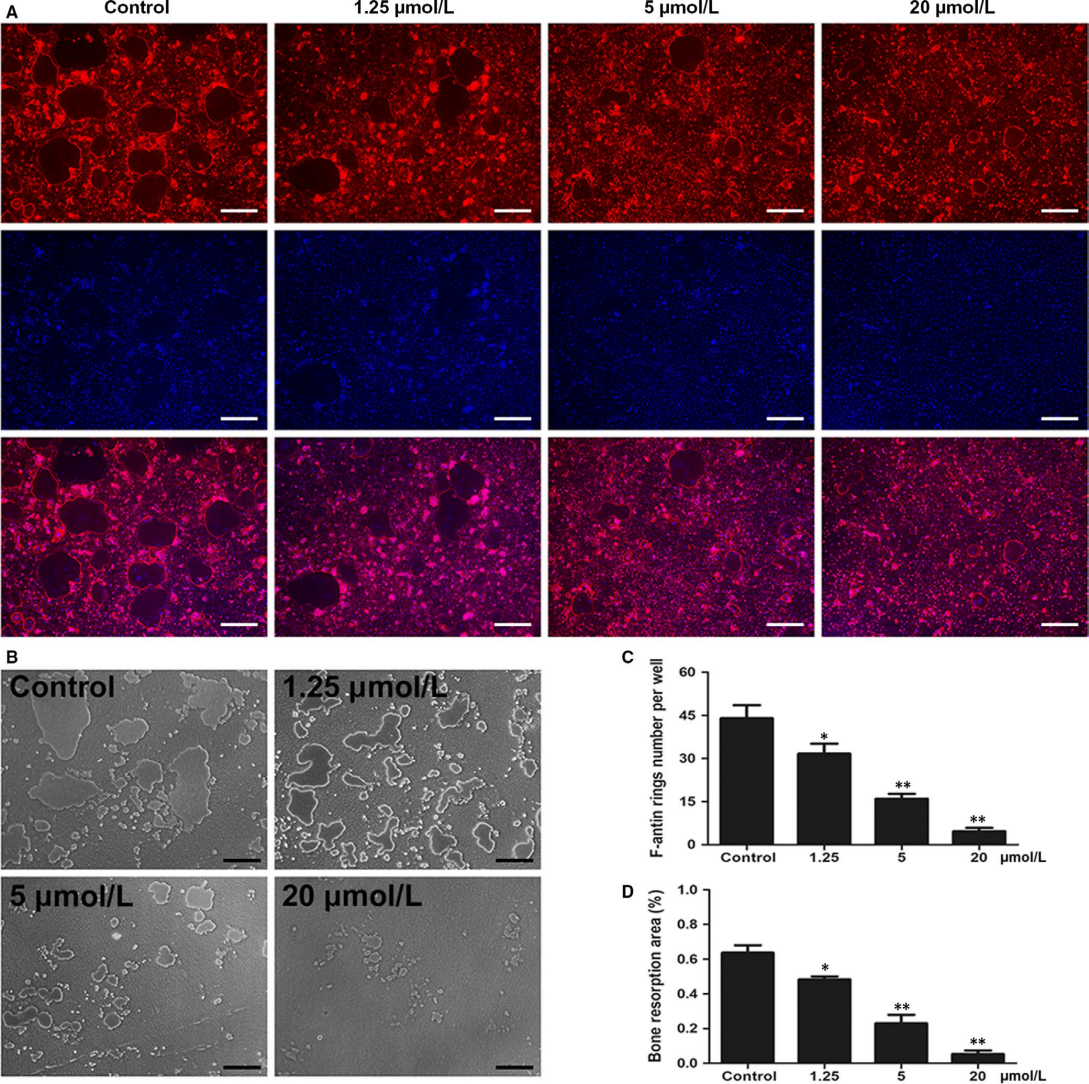
Curcumin suppressed F‐actin ring formation and impaired osteoclastic bone resorption pits in vitro. A, BMMs were induced and cultured in α‐MEM medium with the addition of M‐CSF (30 ng/mL), RANKL (100 ng/mL) and 0, 1.25, 5 or 20 μmol/L curcumin for 6 d; then, the cells were stained with phalloidin and DAPI. The size and number of osteoclasts were observed using a fluorescence microscope. B, BMMs were seeded on Osteo Assay Plate (OAP; Corning) and incubated in osteoclast induction medium for 4 d. When typical osteoclasts were formed on day 4, the osteoclast induction medium containing various curcumin concentrations (0, 1.25, 5 or 20 μmol/L) was replaced and cultured for an additional 2 d. Representative images of bone resorption pits were observed and taken using a light microscope. C, The number of F‐actin rings per well was quantified. D, Percentage of bone resorption area was quantified. Data are presented as mean ± SD; *P < .05 and **P < .01 compared with the control group. Scale bar = 200 μm

Previous studied have indicated that typical F‐actin rings indicate the functional state of osteoclasts and reflect the cytoskeletal integrity of osteoclasts.^21^ Thus, we performed fluorescent staining to evaluate the potential effects of curcumin on F‐actin rings and osteoclast fusion. The results showed that curcumin markedly attenuated the formation of F‐actin rings as the curcumin concentration increased, whereas numerous well‐organized F‐actin rings were observed without curcumin treatment (Figure 2A,C).

Because bone resorption capacity directly reflects the function of osteoclasts, hydroxyapatite‐coated 24‐well plates were used in this study.^22^, ^23^ After incubation in osteoclast induction medium for 4 days, the medium was replaced with medium containing 0, 1.25, 5 or 20 μmol/L curcumin, and the cells were cultured for an additional 2 days. The area of bone resorption significantly increased without curcumin intervention in the control group; however, the formation of osteoclastic resorption pits decreased as the curcumin concentration increased (Figure 2B,D). Therefore, the results showed that the administration of curcumin interfered with the functional state of osteoclasts.

### 3.3 Curcumin ameliorated the up‐regulation of osteoclastic‐related genes in vitro

**Figure 3:**
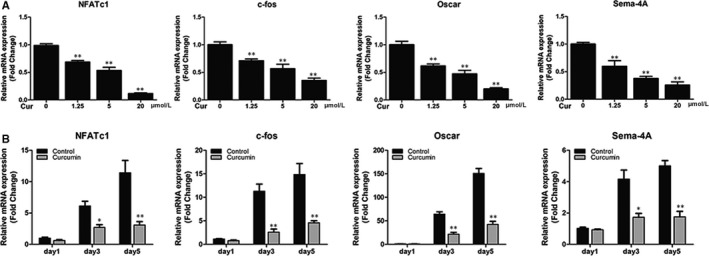
Curcumin down‐regulated the expression of RANKL‐induced osteoclast‐related genes including NFATc1, c‐fos, Oscar and Sema‐4A in vitro. A, BMMs were cultured and induced in osteoclast induction medium containing various curcumin concentrations (0, 1.25, 5 or 20 μmol/L) for 5 d. B, BMMs were incubated in osteoclast induction medium with or without 20 μmol/L curcumin for 1, 3 or 5 d. The expression of osteoclast‐related genes was quantified by RT‐PCR. Data are presented as mean ± SD; *P < .05 and **P < .01 compared with the control group

The expression of osteoclastic‐related genes such as c‐fos, NFATc1, Oscar and Sema‐4A was analysed by RT‐PCR. After pre‐treatment with RANKL, the mRNA levels of osteoclastic‐related genes were significantly stimulated and up‐regulated. However, all four genes were down‐regulated following curcumin intervention in concentration‐ and time‐dependent manners (Figure 3A,B). The results of RT‐PCR were very consistent with the results of TRAP staining and the osteoclast function assays and further confirmed the inhibitory effects of curcumin on osteoclast function.

### 3.4 Immunomodulatory effect of curcumin on macrophage polarization in RAW264.7 cells

**Figure 4:**
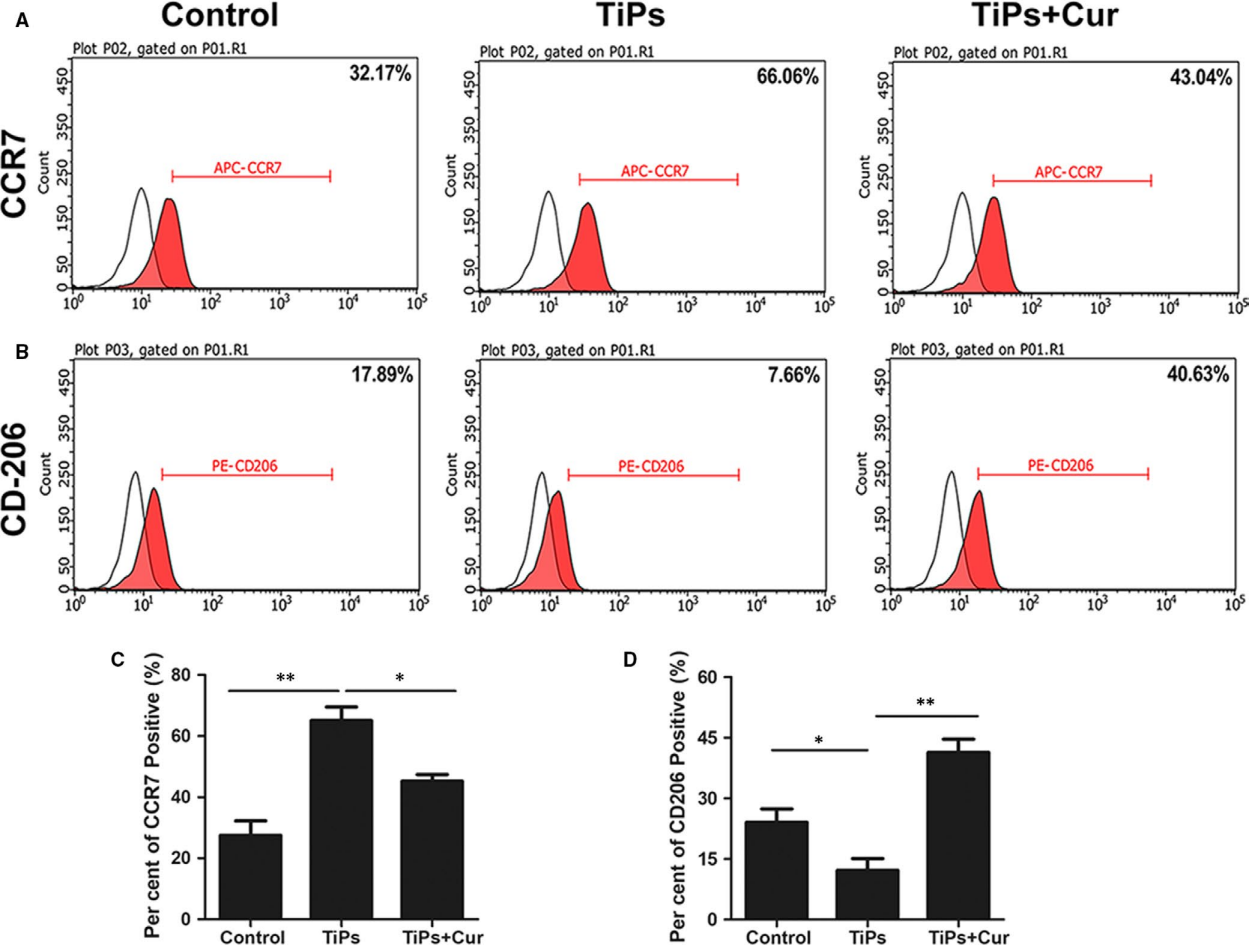
Curcumin regulated TiP‐stimulated macrophage polarization. A, Representative histograms of the M1 marker CCR7 of RAW264.7 cells analysed by flow cytometry. B, Representative histograms of the M2 marker CD206 of RAW264.7 cells analysed by flow cytometry. C, Percentage of CCR7 positive cells was quantified. D, Percentage of CD206 positive cells was quantified. Data are presented as mean ± SD; *P < .05 and **P < .01 compared with the control group

**Figure 5:**
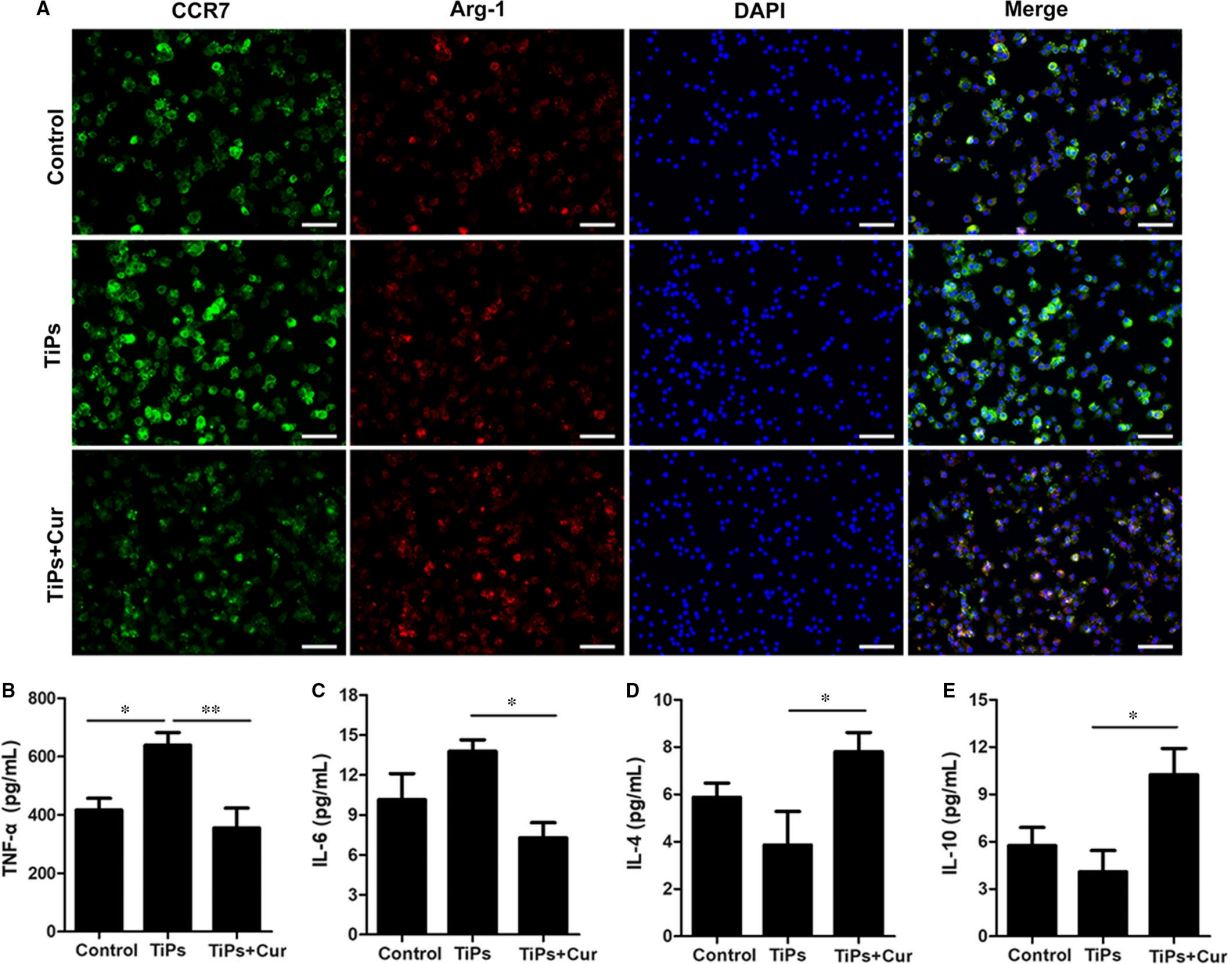
Immunofluorescence staining and ELISA determination of cytokines. A, RAW264.7 cells were plated and cultured in the control, TiPs and TiPs + Cur groups for 3 d. The cells were prepared for immunofluorescence staining. CCR7 (green) indicates M1 macrophages; Arg‐1 (red) indicates M2 macrophages; and nuclei are stained with DAPI (blue). B‐E, ELISA results of cytokines TNF‐α, IL‐4, IL‐6 and IL‐10 by RAW264.7 cells. Data are presented as mean ± SD; *P < .05 and **P < .01 compared with the control group. Scale bar = 50 μm

Recent studies have demonstrated the immunomodulatory effect of macrophage polarization on the regulation of inflammation reactions, leading to alterations in wear debris‐induced osteolysis and bone loss.^8^, ^24^ Therefore, the immunomodulatory effects of curcumin on RAW264.7 cells were investigated using flow cytometry, ELISA, and immunofluorescence staining. The results of flow cytometry (Figure 4) showed that the M1‐type macrophages decreased from 66.06% in the TiPs group to 43.04% in the TiPs + Cur group, whereas the percentage of M2‐type macrophages was higher in the TiPs + Cur group (40.63%) than in the TiPs group (17.89%) and control group (7.66%). In addition, the results of immunofluorescence staining were highly consistent with the results of flow cytometry (Figure 5A). The expression of the M1 marker CCR7 was higher in the TiPs group than in the other groups; however, the M2 marker Arg‐1 was significantly induced by curcumin treatment. Furthermore, the secretion levels of proinflammatory and anti‐inflammatory cytokines were measured by ELISA. The results indicated that the M1 cytokines TNF‐α and IL‐6 were markedly stimulated by TiPs treatment without curcumin, whereas the production of M2 cytokines (IL‐4 and IL‐10) was higher with curcumin intervention than that in the other groups (Figure 5B‐E). Collectively, these results indicated that curcumin has an immunomodulatory ability in RAW264.7 cells to regulate macrophage polarization.

### 3.5 Curcumin alleviates osteoclast differentiation and functions by inhibiting the Akt and NF‐κB pathways

**Figure 6:**
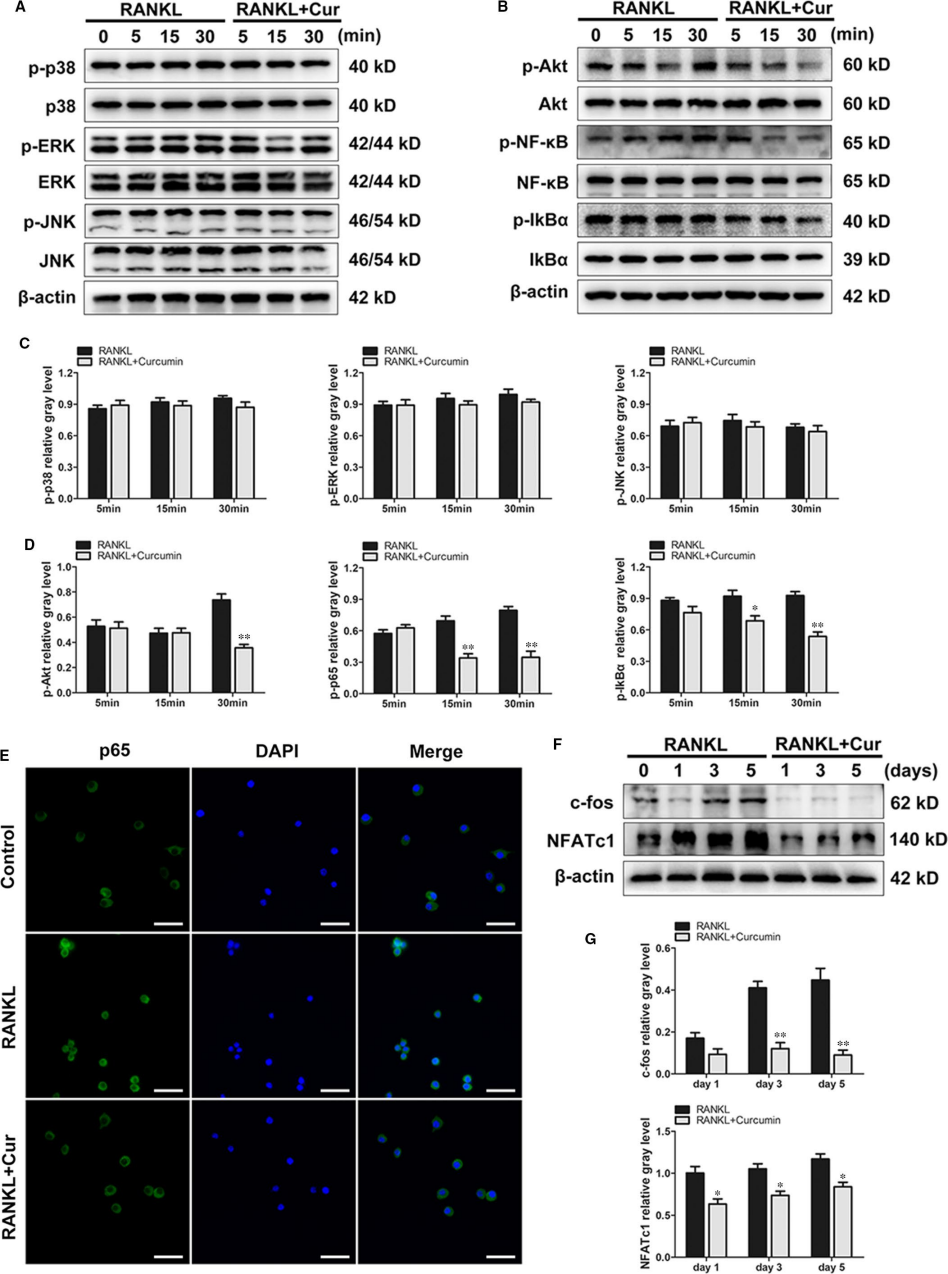
Curcumin ameliorated the activation of Akt and NF‐κB p65 phosphorylation but had no inhibitory effect on the MAPK pathways. (A and B), RAW264.7 cells were pretreated with or without curcumin for 4 h and then with 100 ng/mL RANKL for indicated time periods (0, 5, 15 or 30 min). Then, the cells were collected and lysed for Western blot analysis. C, The relative grey levels corresponding to p‐ERK, p‐JNK and p‐p38 were quantified and were normalized to β‐actin using ImageJ software. D, The relative grey levels corresponding to p‐Akt, p‐p65 and p‐IκBα were quantified and normalized to β‐actin using ImageJ software. E, RAW264.7 cells were pretreated with or without 20 μmol/L curcumin for 4 h and then stimulated by RANKL for 30 min. The cells were prepared for immunofluorescence staining of p65. F, RAW264.7 cells were cultured in osteoclast induction medium with or without 20 μmol/L curcumin for 1, 3 or 5 d. Cells were then collected and lysed for Western blot analysis. G, The relative grey levels corresponding to c‐fos and NFATc1 were quantified and normalized to β‐actin using ImageJ software. Data are presented as mean ± SD; *P < .05 and **P < .01 compared with the control group. Scale bar = 100 μm

The potential mechanisms of this inhibitory effect on RANKL‐stimulated osteoclast differentiation and functions were further explored by Western blotting. Numerous studies have suggested that several osteoclastic signalling pathways, including the MAPKs, NF‐κB and PI3k/Akt pathways, were associated with the activation and differentiation of osteoclast precursor cells.^25^, ^26^, ^27^ We first investigated the subfamilies of MARK pathways (ERK, JNK and p38 pathways), and the results suggested that curcumin had no inhibitory effect on the activation of MARK pathways at all time‐points (Figure 6A,C). However, an inhibitory effect of curcumin treatment on Akt phosphorylation was observed at 30 minutes. Furthermore, curcumin also alleviated the phosphorylation of the p65 and IκBα (NF‐κB) pathways at the 15 and 30 minutes time‐points, indicating that curcumin exerted an inhibitory effect on osteoclast differentiation by ameliorating the up‐regulation of Akt and NF‐κB phosphorylation (Figure 6B,D). Immunofluorescence staining of p65 further verified this inhibitory effect on the NF‐κB pathway (Figure 6E).

In addition, two downstream transcription factors, c‐fos and NFATc1, were investigated. BMMs were plated and incubated with or without curcumin intervention for 1, 3 and 5 days. The results indicated that downstream transcription factors were markedly down‐regulated with curcumin treatment (Figure 6F,G). Therefore, the results of the investigations of the osteoclastic mechanisms revealed that curcumin attenuated RANKL‐mediated osteoclast formation by modulating macrophage polarization and further inhibiting the activation of the Akt/NF‐κB/NFATc1 pathways.

### 3.6 Curcumin exerted a protective effect on TiP‐stimulated bone destruction

**Figure 7:**
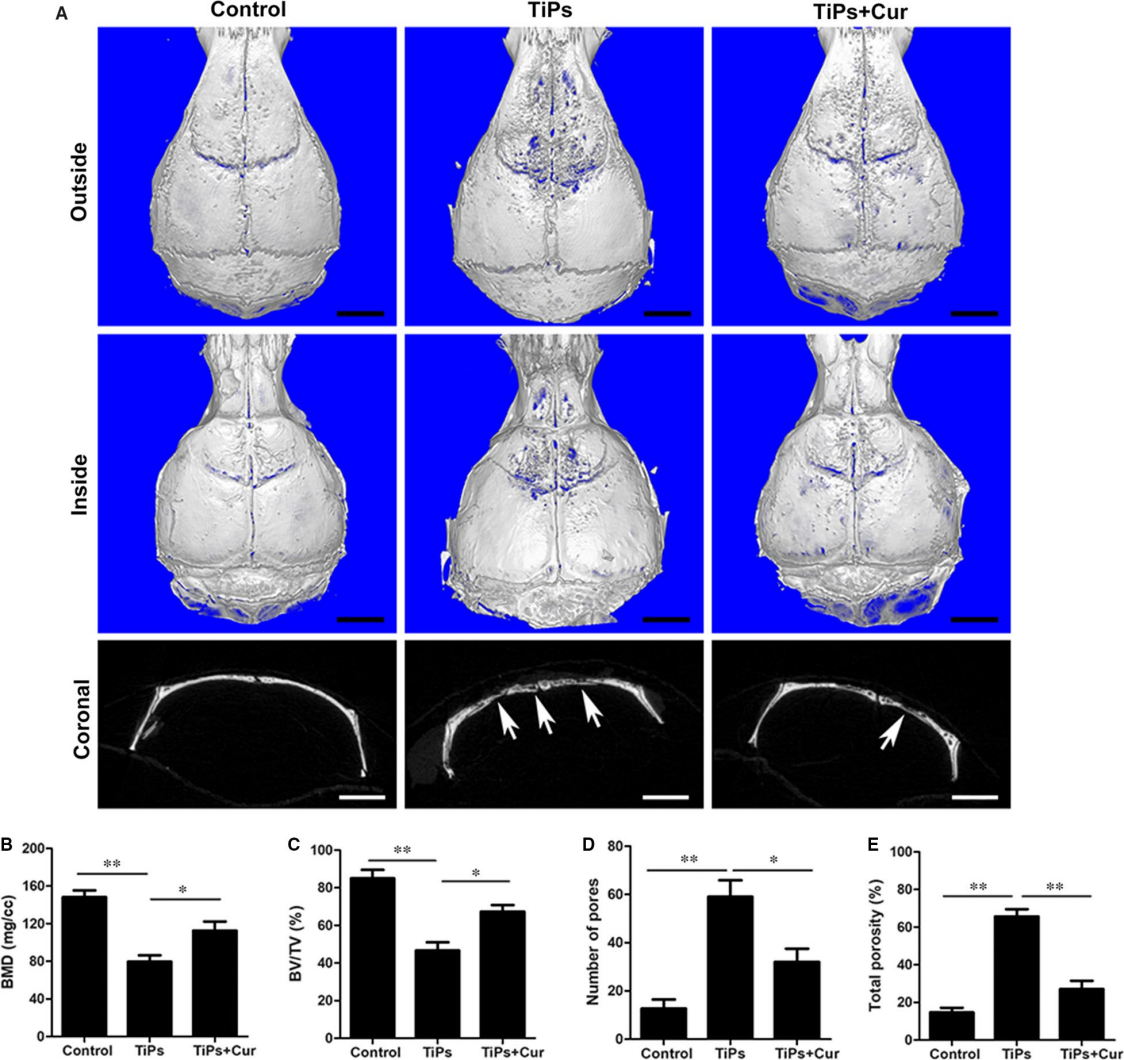
Curcumin attenuated TiP‐induced mouse calvarial osteolysis in vivo. A, Representative micro‐CT 3D and 2D reconstructed images of the calvaria in each group. The white arrows indicate bone loss. B, BMD, (C) BV/TV, (D) total porosity and (E) number of pores of each group were measured. Data are presented as mean ± SD; *P < .05 and **P < .01 compared with the control group. Scale bar = 2 mm

A calvarial resorption model was established to assess the therapeutic effectiveness of curcumin for treating TiP‐stimulated osteolysis in vivo*,* and the characteristics of TiPs are shown in Figure S2. The reconstruction images from micro‐CT are presented in Figure 7A. The results showed clear bone destruction and resorption following TiPs treatment. However, curcumin significantly alleviated the degree of bone destruction and bone loss in the TiPs + Cur group. The white arrows indicate bone resorption and destruction. As shown in Figure 7B‐E, the micro‐CT data further confirmed that BMD and BV/TV were significantly decreased, and the total porosity and number of pores were markedly increased with TiPs intervention. After curcumin treatment for 2 weeks, BMD and BV/TV markedly increased, whereas the total porosity and number of pores decreased in mouse calvariae, indicating that curcumin exerted a therapeutic effect in osteolysis mice.

**Figure 8:**
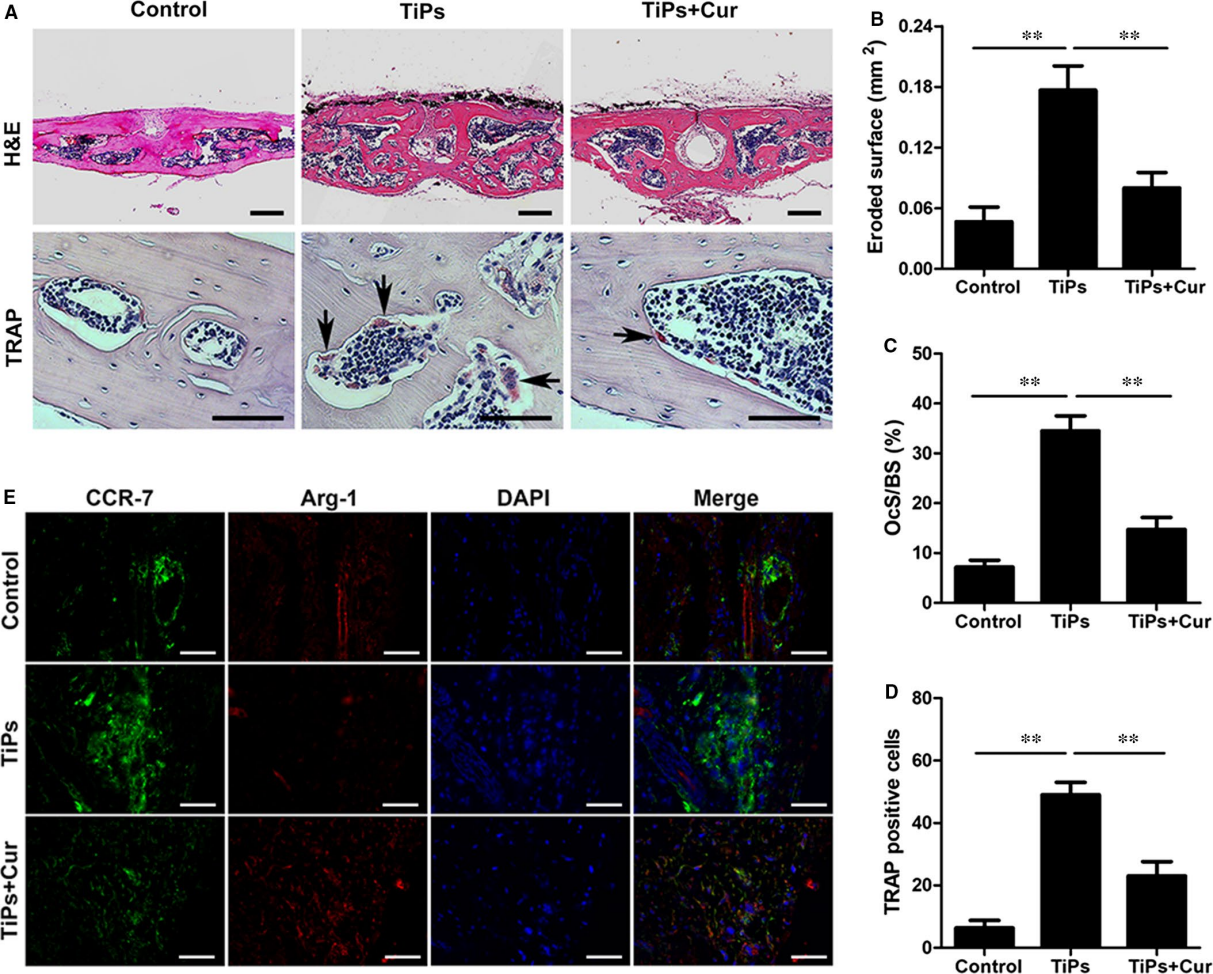
Curcumin protected TiP‐stimulated osteolysis and modulated macrophage polarization in vivo. A, Representative histological images of H&E staining (Scale bar = 100 μm) and TRAP staining (Scale bar = 50 μm). TRAP‐positive cells are indicated by black arrows. B, Eroded surface area, (C) OcS/BS and (D) the number of TRAP‐positive cells of each group were measured using Image‐Pro Plus software 6.0. E, Images of immunofluorescence staining on the skin of head. CCR7 (M1 marker)‐positive cells were stained in green, and Arg‐1 (M2 marker)‐positive cells were stained in red; nuclei were stained in blue (Scale bar = 50 μm). Data are presented as mean ± SD; *P < .05 and **P < .01 compared with the control group

Histological evaluations were performed to verify the inhibitory effect of curcumin on wear debris‐mediated osteolysis in mice. The H&E results indicated that curcumin attenuated the severity of bone loss and inflammatory reactions induced by TiPs. Numerous typical osteoclasts were detected following TiPs stimulation (indicated by black arrows); however, curcumin markedly reduced the number of osteoclasts (Figure 8A). The results of the assessments of the eroded surface, number of TRAP‐positive cells and OCs/BS were consistent with the results of the micro‐CT and histological evaluations (Figure 8B‐D). Furthermore, immunochemical staining of p65 in vivo further confirmed that the expression of p65 was significantly increased in the TiPs group, whereas p65 expression was clearly inhibited in the curcumin‐treated group compared with the TiPs group (Figure S1).

Immunofluorescence staining of CCR‐7 and Arg‐1 was also performed to assess macrophage polarization in vivo. The results showed that the expression of the M1 marker CCR‐7 (green) was clearly increased by TiPs stimulation in the positive control group, whereas the fluorescence intensity of the M2 marker Arg‐1 (red) was the lowest in the TiPs group. However, the TiPs + Cur group had a lower proportion of CCR‐7 (M1) macrophages and a higher proportion of Arg‐1 (M2) macrophages following curcumin intervention (Figure 8E). Thus, the results of macrophage polarization in mice were very consistent with these results in vitro.

## 4 DISCUSSION

Numerous studies have shown that the balance between osteoblast‐induced osteogenesis and osteoclast‐stimulated bone destruction is essential for the maintenance of bone homeostasis and that excess osteoclastic activity and activation lead to bone diseases such as osteolysis and osteoporosis.^28^, ^29^, ^30^ Thus, it is necessary to inhibit osteoclast activity and formation to treat bone‐loss‐related diseases. Curcumin, a major biologic component of *Curcuma longa* with anti‐inflammatory and antioxidant properties, has been shown to exhibit therapeutic effectiveness in inflammatory diseases and exert an immunomodulatory effect on macrophage polarization.^12^ In our previous study, we verified the protective property of curcumin against polymethylmethacrylate‐induced osteolysis and bone destruction in vivo.^16^ However, the immunomodulatory and direct anti‐osteoclastogenesis effects on RANKL‐mediated osteoclast formation in vitro have not been explored, and the potential molecular and cellular mechanisms of this inhibitory effect have not been clarified.

Previous studies demonstrated that inflammatory responses and the release of cytokines were necessary, in different manners, to stimulate and activate the initiation, recruitment, differentiation and maturation of osteoclast precursor cells.^31^, ^32^ Previous studies suggested that proinflammatory cytokines enhanced the binding of RANKL to RANK, which is a receptor on the cell membranes of osteoclast precursor cells. After RANLK binds to RANK, the classic osteoclastic pathways such as the MAPKs, Akt and NF‐κB are further activated and eventually activate c‐fos and NFATc1.^33^, ^34^, ^35^ The NF‐κB pathway, which is one of the primary osteoclast formation pathways, consists of a p65 homodimer and a p50/p65 heterodimer.^30^, ^36^ Following activation, the active form of NF‐κB is induced and separates from the inhibitor IκB, and it then enters the nucleus and regulates the activation of NFATc1.^37^ The Akt pathway is another important signalling pathway that induces the formation of mature osteoclasts and the expression of osteoclastic genes.^38^ It has been shown that wear debris is not able to stimulate the differentiation of osteoclast precursor cells in the absence of RANKL modulation. As a master regulator of osteoclastogenesis, NFATc1 enhances the expression of osteoclastic‐related genes and initiates osteoclast precursor cell differentiation.^39^, ^40^ Without the activation of NFATc1, however, RANKL may not completely induce the differentiation of BMMs. In contrast, the ectopic expression of NFATc1 was found to regulate osteoclast precursor cell differentiation without RANKL stimulation.^41^, ^42^ NFATc1 may induce osteoclast formation and gene expression independent of RANKL. Therefore, inhibiting the release of proinflammatory cytokines and blocking the osteoclastic signalling pathways may represent effective targets for therapeutic agents. In our study, we demonstrated that curcumin ameliorated the activation of Akt and NF‐κB p65 phosphorylation but had no effect on ERK, JNK and p38 phosphorylation, indicating that curcumin treatment had no inhibitory effect on the MAPK pathways. The decrease in IκBα phosphorylation further confirmed that the NF‐κB pathway was blocked following curcumin intervention. In addition, c‐fos and NFATc1, two downstream transcription factors, were also markedly decreased at the gene and cellular levels following curcumin treatment.

**Figure 9:**
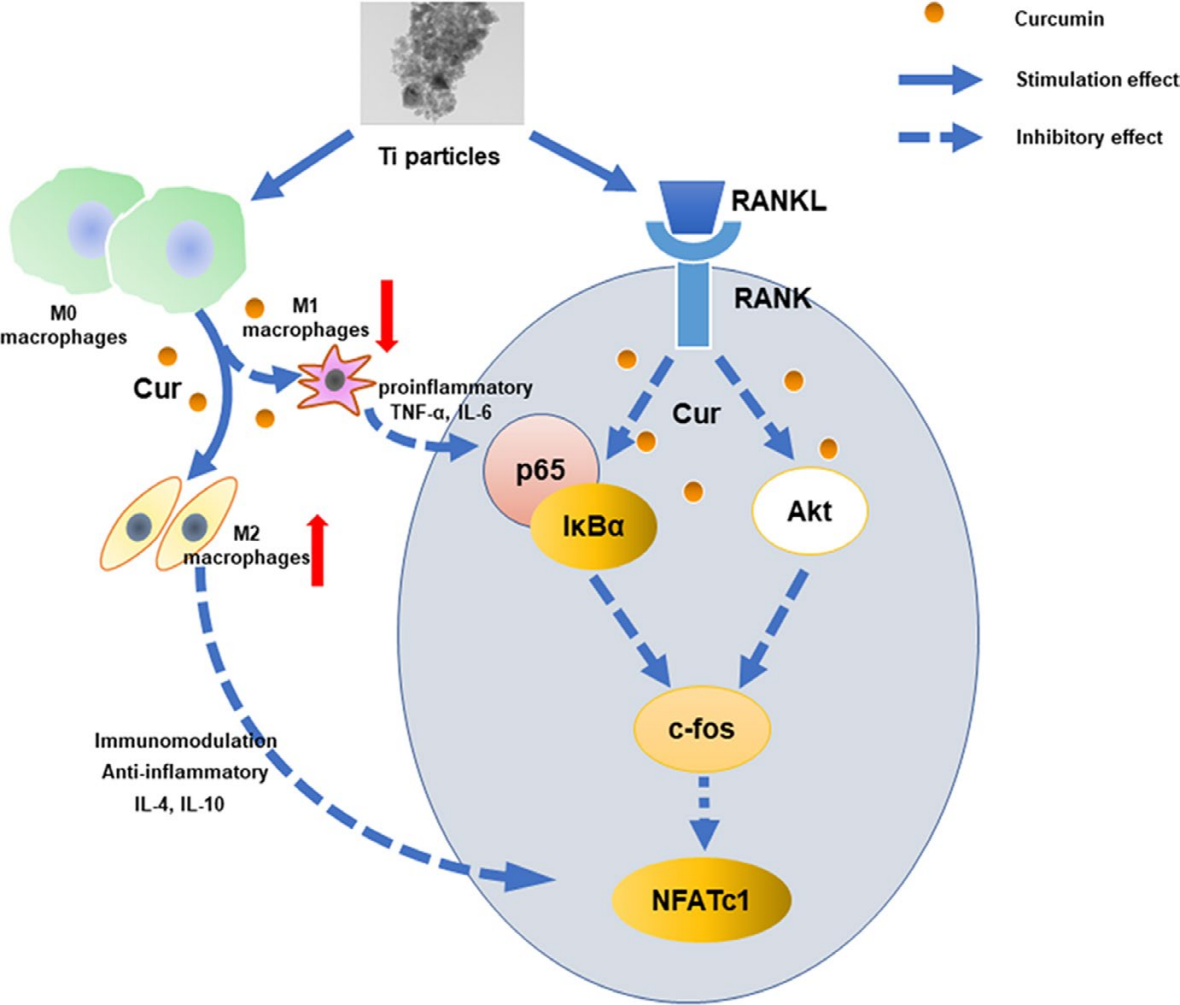
Schematic illustration of curcumin has immunomodulatory and inhibitory effects on RANKL‐induced osteoclast formation. Curcumin attenuated the up‐regulation of Akt and NF‐κB p65 phosphorylation and the activation of the downstream transcription factor NFATc1. In addition, curcumin created an immunomodulatory microenvironment and promoted macrophage polarization from the M1‐type to the M2‐type phenotype

Because the state of macrophage polarization is critical for the inflammatory microenvironment, the immunomodulatory effect of curcumin was also evaluated. Wang et al reported that probiotic treatment protected against CoCrMo particle stimulated osteolysis in mice by regulating the M1/M2 ratio.^8^ Li et al reported that deacylcynaropicrin inhibited RANKL‐mediated osteoclast fusion by promoting M2‐type macrophage polarization.^24^ A large number of studies have reported that M1‐type macrophages were stimulated and induced by wear debris, which is generated from the surface of an implant prosthesis, and released proinflammatory cytokines, creating a proinflammatory microenvironment.^10^, ^43^, ^44^ This proinflammatory microenvironment further activates the osteoclastic signalling pathways and promotes the recruitment and differentiation of osteoclast precursor cells.^8^ In our study, following TiPs stimulation, the M1‐specific marker CCR7 was significantly increased, whereas the M2‐specific markers CD206 and Arg‐1 were markedly decreased. However, curcumin decreased the proportion of M1‐type macrophages and promoted the polarization of macrophages from the M1‐type to the M2‐type phenotype. The ELISA results further verified that curcumin reversed the TiP‐stimulated up‐regulation of proinflammatory cytokines and the down‐regulation of anti‐inflammatory cytokines. Therefore, the immunomodulation of macrophage phenotypes and the microenvironment represents a potential therapeutic approach to ameliorate wear debris‐mediated osteolysis (Figure 9).

As expected, curcumin had a protective effect on RANKL‐mediated osteoclastogenesis by promoting macrophage polarization from the M1‐type to the M2‐type phenotype and attenuating the activation of the Akt and NF‐κB pathways. However, our study had several limitations. First, our findings indicated that curcumin exerted an immunomodulatory effect on macrophage polarization by down‐regulating proinflammatory cytokines and up‐regulating anti‐inflammatory cytokines. However, the potential mechanisms by which curcumin mediates this change in the macrophage functional state remain unknown. Therefore, further studies on this change in macrophage phenotype may enhance our understanding of the protective effect of curcumin on wear particle‐induced osteolysis. Second, although the proinflammatory cytokines mainly released by macrophages and the consequent immune responses change the local immune status, various cells, including osteoblasts, fibroblasts and mesenchymal cells, may also release proinflammatory cytokines and participate in the process of wear particle‐mediated osteolysis. The effectiveness and safety of curcumin in other cells will be investigated in our future studies.

To conclude, our results suggested that curcumin ameliorated the RANKL‐mediated differentiation, fusion and maturation of osteoclasts and had an immunomodulatory effect on macrophage polarization. Examinations of the potential molecular and cellular mechanisms revealed that this protective effect of curcumin on osteoclastogenesis was mediated by attenuating the up‐regulation of Akt and p65 phosphorylation and the activation of the downstream transcription factor NFATc1. Using an in vivo mouse calvarial destruction model, it was further confirmed that curcumin ameliorated TiP‐stimulated osteolysis and bone loss, thereby demonstrating its potential therapeutic agent for the treatment of osteoclastic disease.

## CONFLICT OF INTEREST

The authors declare no conflict of interest.

## AUTHOR CONTRIBUTIONS

Tao Cheng, Xianlong Zhang and Yebin Qian designed the study. Chao Yang and Kechao Zhu contributed equally to this study. Chao Yang analysed the data and drafted the manuscript. Xiangwei Yuan, Yebin Qian, Xianlong Zhang and Tao Cheng helped revise the manuscript. All authors reviewed the manuscript and approved the final manuscript. All authors read and approved the final manuscript.

## Data Availability

All data used to support the findings of this study are available from the corresponding authors’ request.
