# Supplementary material for: Curcumin has immunomodulatory effects on RANKL‐stimulated osteoclastogenesis in vitro and titanium nanoparticle‐induced bone loss in vivo
Source: J Cell Mol Med. 2019 Dec 17;24(2):1553–67. doi: 10.1111/jcmm.14842 (PMC6991655; doi:10.1111/jcmm.14842)
Supplement: Supplementary file 1 [file JCMM-24-1553-s001.docx]

**Curcumin has immunomodulatory effects on RANKL-stimulated osteoclastogenesis *in vitro* and titanium nanoparticle-induced bone loss *in vivo***

Chao Yang^1^, Kechao Zhu^1^, Xiangwei Yuan^1^, Xianlong Zhang^1^, Yebin Qian^1^ and Tao Cheng^1^

^1^ Department of Orthopedics, Shanghai Jiao Tong University Affiliated Sixth People’s Hospital, Shanghai 200233, China

**Correspondence**

Xianlong Zhang

Email: dr_[zhangxianlong@163.com](mailto:zhangxianlong@163.com)

Yebin Qian

Email: [yebinqian@aliyun.com](mailto:yebinqian@aliyun.com)

Tao Cheng

Email: [dr_tao.cheng@hotmail.com](mailto:dr_tao.cheng@hotmail.com)

Chao Yang and Kechao Zhu have contributed equally to the work.

Immunochemical staining of p65 in vivo was performed. As shown in **FIGURE S1**, the expression of p65 was significantly increased in the TiP-treated group, whereas p65 expression was clearly inhibited in the curcumin-treated group.


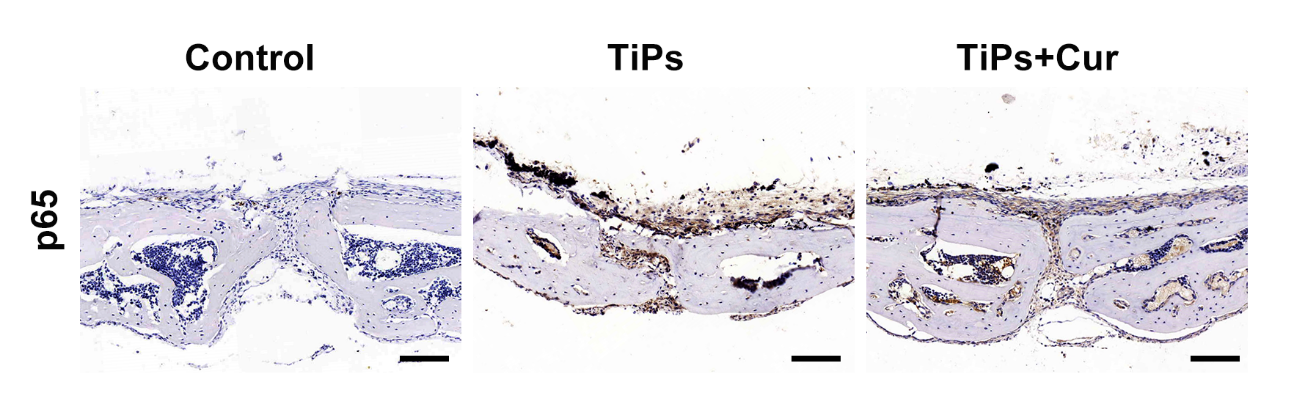


**FIGURE S1.** Immunohistochemical staining for p65 in mouse calvarial of each group. Scale bar = 100 μm

The characteristics of titanium particles (TiPs) were evaluated by transmission electron microscope (TEM). The morphology of the TiPs was observed by TEM. The characteristics of TiPs were presented in **FIGURE S2**. Most TiPs were of irregular morphology and flock together, which were findings similar to the wear particles as previously described.


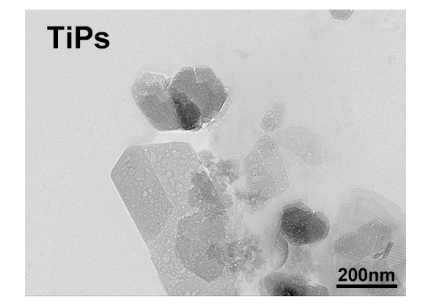


**FIGURE S2.** Characterizations of TiPs were observed by TEM.
